# Blood Biomarkers Reflecting Brain Pathology—From Common Grounds to Rare Frontiers

**DOI:** 10.1002/jimd.70032

**Published:** 2025-05-05

**Authors:** Isabelle Weinhofer, Paulus Rommer, Johannes Berger

**Affiliations:** ^1^ Department Pathobiology of the Nervous System, Center for Brain Research Medical University of Vienna Vienna Austria; ^2^ Department of Neurology, Comprehensive Center for Clinical Neurosciences and Mental Health Medical University of Vienna Vienna Austria

**Keywords:** metachromatic leukodystrophy, multiple sclerosis, neurodegeneration, neurofilament light chain protein, neuroinflammation, X‐linked adrenoleukodystrophy

## Abstract

Understanding pathological changes in the brain is essential for guiding treatment decisions in brain injuries and diseases. Despite significant advances in brain imaging techniques, clinical practice still faces challenges due to infrastructure reliance and high resource demands. This review explores the current knowledge on blood‐based biomarkers that indicate brain pathology, which can assist in identifying at‐risk patients, diagnosing patients, predicting disease progression, and treatment response. We focus on the inherited metabolic disorders X‐linked adrenoleukodystrophy (X‐ALD) and metachromatic leukodystrophy (MLD) which share remarkable phenotypic variability. Disease‐specific increases in the lipid metabolites lyso‐PC26:0 in X‐ALD and sulfatides in MLD might contribute to predicting clinical manifestation. Disease‐unspecific biomarkers for axonal damage (neurofilament light chain protein, NfL) and glial degeneration (glial fibrillary acidic protein, GFAP) are able to distinguish X‐ALD and MLD phenotypes at the group level. The implementation of X‐ALD into newborn screening programs in various countries, including several U.S. states, has increased the demand for predictive blood‐based biomarkers capable of detecting the early conversion from the pre‐symptomatic to the early neuroinflammatory cerebral form of X‐ALD. Among different biomarkers, NfL has proven most effective in reflecting neuroinflammation and correlating with brain lesion volume and the magnetic resonance imaging (MRI)‐based severity scores. We discuss how NfL has moved from initial proof‐of‐principle towards proof‐of‐concept studies in brain disorders such as multiple sclerosis and how this knowledge could be applied for the clinical implementation of NfL in rare inherited metabolic disorders such as X‐ALD.

## 1 Introduction

Brain pathology can result from many different sources, including traumatic brain injury, stroke, or cancer. In addition, it may arise from common neurodevelopmental or neurodegenerative disorders, including Alzheimer's disease (AD) and multiple sclerosis (MS), or rare inherited disorders such as X‐linked adrenoleukodystrophy (X‐ALD, OMIM #300100) and metachromatic leukodystrophy (MLD, OMIM #250100). For appropriate treatment and care of brain damage, timely and accurate diagnosis in early disease stages is critical. To date, brain imaging techniques such as magnetic resonance imaging (MRI), functional MRI (fMRI), positron emission tomography (PET), or diffusion tensor imaging (DTI) are most widely applied in hospitals to diagnose disease and evaluate brain health. Despite notable advances in imaging techniques like fiber tracking, iron‐specific sequences, improved lesion volumetric analysis, or the development of novel PET tracers, limitations persist in clinical practice, including dependence on infrastructure and high resource requirements, such as those for PET tracers. More recently, optical coherence tomography (OCT) has been shown to be a reliable marker for neurodegeneration in MS and other neurodegenerative disorders but also recently in the rare neurometabolic disease X‐ALD [1, 2, 3]. Nevertheless, OCT has its limitations due to specificity. The recent development of highly sensitive techniques to quantify blood biomarkers for brain damage has ushered in a new era. But how reliably is brain pathology reflected in blood‐based biomarkers, and how can the results be applied in clinical settings? In this review, we (i) discuss the purpose and advantage of blood biomarkers; (ii) focus on the inherited metabolic disorders X‐ALD and MLD and the needs for blood‐based biomarkers; (iii) discuss how well blood biomarkers reflect neurodegeneration and glial damage in more common disorders such as MS; (iv) discuss how such blood biomarkers can be translated to the clinics; (v) present the status quo of current implementation of blood biomarkers for brain pathologies in clinical practice; (vi) examine X‐ALD as a rare inherited brain disorder where leveraging existing biomarker knowledge derived from the more common diseases could expedite the readiness for clinical application; and (vii) give an outlook on possibly emerging fields and technologies with potential to evaluate brain pathologies in blood‐based samples.

## 2 Purpose of Biomarkers and the Advantage of Blood Biomarkers

**FIGURE 1:**
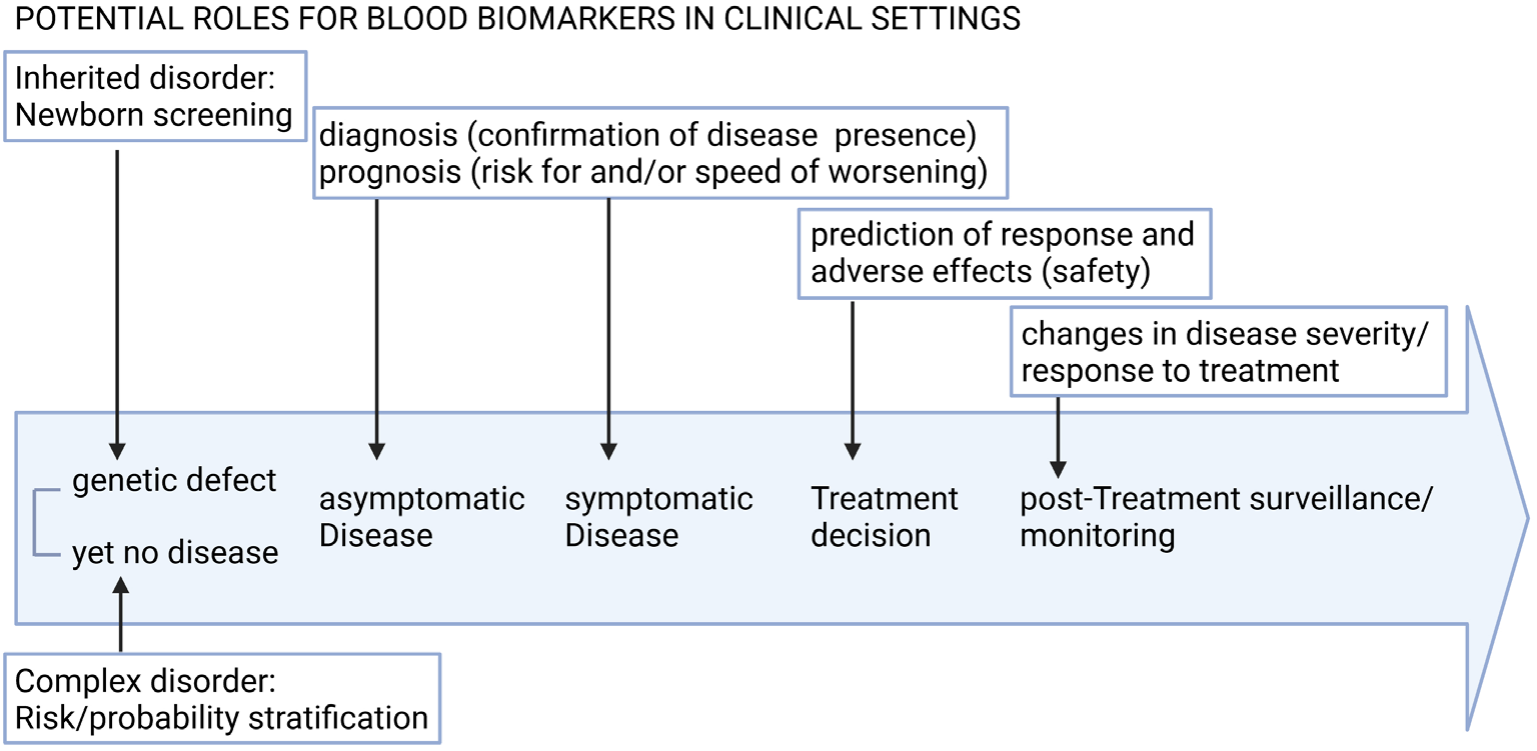
The purposes of blood‐based biomarker usage in complex neurodegenerative and inherited metabolic diseases.

Two decades ago, a biomarker was defined by the National Institutes of Health Biomarkers Definitions Working Group as “a characteristic that is objectively measured and evaluated as an indicator of normal biological processes, pathogenic processes, or pharmacologic responses to a therapeutic intervention” [4]. Accordingly, in clinical practice, biomarkers can serve various purposes that apply to both monogenetic inherited metabolic and complex disorders and include guidance of diagnosis and estimation of disease risk or probability (Figure 1). Beyond diagnosis, biomarkers provide prognostic information, aiding in predicting the likely course and outcome of a disease. Additionally, they are instrumental in monitoring the progression of a disorder, allowing healthcare professionals to assess the efficacy of treatments and make necessary adjustments [5]. Next to imaging biomarkers, cerebral spinal fluid (CSF) biomarkers have also been progressively incorporated into the clinical routine to assess brain health. Despite the advantage of CSF being close to the brain extracellular space and, thus, containing high concentrations of CNS‐derived proteins, assessing biomarkers in the CSF requires an invasive and painful lumbar puncture procedure. For a long time, there was little belief that neurological biomarkers indicating brain damage could leak into the peripheral blood in detectable amounts due to the brain being protected by the blood–brain barrier (BBB). Recent technological advancements, such as the development of single‐molecule array (Simoa) and electrochemiluminescence (ECL)‐based assays, have led to a significant breakthrough in detecting genuine brain changes through peripheral biomarkers. These advancements allow for the highly sensitive detection of brain‐derived markers, even in trace amounts, within the blood. In addition, the advantage of blood biomarkers being easily accessible, cost‐effective, and accurate through ultrasensitive measurement gives them a potential for widespread clinical application [6].

## 3 The Rare Neurometabolic Disorders X‐Linked Adrenoleukodystrophy and Metachromatic Leukodystrophy

With a combined incidence of 1:14700 newborns, the neurodegenerative disorder X‐ALD is the most common monogenetically inherited leukodystrophy [7, 8]. X‐ALD is caused by mutations in the X‐chromosomal *ABCD1* gene, which encodes a transporter for the peroxisomal import of saturated CoA‐activated very long‐chain fatty acids (VLCFA; ≥ C22:0) destined for degradation by peroxisomal β‐oxidation [9]. ABCD1 deficiency leads to the accumulation of VLCFAs in body fluids and tissues, most notably in brain white matter, the spinal cord, and the adrenal cortex [7]. Clinically, X‐ALD presents with remarkable phenotypic variability, with the core syndrome being a slowly progressive dying‐back axonopathy in the spinal cord, typically of young adulthood onset, referred to as adrenomyeloneuropathy (AMN). AMN will affect almost all male X‐ALD patients [10], and the myeloneuropathy affects about 80% of female X‐ALD patients [11]. With peak onset in early boyhood, around 60% of male X‐ALD patients develop a rapidly progressive inflammatory cerebral demyelination known as cerebral ALD (CALD), resulting in the destruction of myelin and axons with concurrent BBB leakiness and infiltration of pro‐inflammatory skewed monocytes/macrophages and T cells [12, 13, 14]. As several members of a family with the same *ABCD1* mutation can present with the entire clinical spectrum of X‐ALD [15] and even monozygotic twins can display highly variable clinical manifestations [16], no general genotype–phenotype correlation exists in X‐ALD [8]. Although the amount of VLCFAs is a valid blood‐based biomarker for the diagnosis of X‐ALD, total plasma VLCFA levels are not predictive of clinical outcomes. However, a recent study by Jaspers and colleagues demonstrated that plasma lysophosphatidylcholine C26:0 (lyso‐PC26:0) is significantly increased in cerebral X‐ALD patients compared to pure AMN patients at the group level and thus might hold some potential for future applications [17]. In their study, the authors also included three X‐ALD patients before and after onset of CALD and found that before the start of CALD, all three patients presented with lyso‐PC26:0 levels in the upper range of X‐ALD patients without CALD [17]. Although VLCFAs have previously been linked to pro‐inflammatory activation in innate immune cells [14], this study, for the first time, provides strong evidence of a direct pathogenic effect of the VLCFA accumulation in X‐ALD. These findings are important for the consideration of VLCFA‐lowering treatments in X‐ALD.

In CALD patients, MRI abnormalities reflecting progressive white matter lesions precede the onset of functional neurological deficits such as behavioral decline, visual and auditory dysfunction, gait abnormalities, incontinence, and seizures [18]. This pre‐symptomatic period provides a critical therapeutic “window of opportunity,” emphasizing the need for early and accurate diagnosis to initiate treatment regimens [18]. If detected in these earliest stages, CALD can be stopped by hematopoietic stem cell transplantation (HSCT) or gene therapy. With regard to gene therapeutic approach, it should be mentioned that two recent papers reported haematologic cancer after gene therapy for CALD [19, 20].

Missing the early disease stage renders both HSCT and gene therapy ineffective in halting CALD, leading to premature death or a vegetative state in affected patients. Also, pharmacological strategies currently investigated to halt neuroinflammation, demyelination, and neuronal cell death, such as Leriglitazone, seem to require treatment at early disease stages in order to be successful [21]. Therefore, X‐ALD has been added to newborn screening (NBS) in the U.S.A., Taiwan, and The Netherlands, with evaluation trials underway in other countries. In these screening approaches, the diagnosis of X‐ALD is based on elevated lyso‐PC26:0 levels in newborn dried blot spots. A retrospective follow‐up study of the Minnesota NBS from February 2017 to February 2022 compared clinical symptoms in young children diagnosed with X‐ALD (median age 5.0 years [2.7–7.7]) with their initial NBS lyso‐PC26:0 levels and found higher levels in those who developed adrenal insufficiency, CALD, or both [22]. Despite the early time point (several of the children in the asymptomatic group will likely later develop CALD), the data are very promising and indicate that consistently timed neonatally collected lyso‐PC26:0 levels might identify patients at increased risk for early‐onset disease [22]. This study also compared diagnostic VLCFA values with NBS lyso‐PC26:0 values and found that NBS lyso‐PC26:0 provided a better discrimination between early symptomatic cases and still asymptomatic cases (median age: 5 years).

These findings suggest that the NBS lyso‐PC26:0 measurements may be superior to plasma VLCFA as a predictive blood biomarker for stratifying clinical disease risk. The results of the 10‐ and 15‐year follow‐up studies will further clarify this aspect. Another interesting observation from the study was that Lyso‐PC26:0 levels in asymptomatic X‐ALD boys were comparable to those of detected heterozygous female carriers who only extremely rarely develop CALD. Over the five‐year period of the Minnesota NBS, 323314 newborns were screened, leading to the diagnosis of X‐ALD in 32 males and 11 females. This corresponds to an incidence of 1 in 7519 newborns, which is significantly higher than earlier estimates of 1 in 14 700 [7, 8, 23].

The NBS has also highlighted the possibility of *ABCD1* gene variants of uncertain significance. Such hypomorphic mutations possibly resulting in reduced but not absent protein function were previously unknown in X‐ALD but have been identified in other disorders. It remains to be determined whether these variants of uncertain significance are pathogenic, as this is essential for adjusting the cut‐off levels to prevent the identification of non‐disease variants [24]. As described by Engelen and Kemp [25], global initiatives such as the Grey Zone project, initiated by the nonprofit organization ALD Connect, are required to improve the understanding of variants of uncertain significance and to assess their potential pathogenicity.

A common well‐known example of a disorder associated with hypomorphic mutations is the arylsulfatase A (ARSA) pseudodeficiency allele, which causes reduced activity of the lysosomal ARSA protein but does not lead to symptoms or clinical features of MLD [26]. Like X‐ALD, the lysosomal storage disease MLD is a rare genetic neurometabolic disorder that affects the central and peripheral nervous systems, leading to demyelination, neuronal dysfunction, and neurodegeneration [27]. On a molecular level, MLD is caused by mutations in the ARSA gene that encodes an enzyme involved in the breakdown of sulfatides within lysosomes. Accordingly, loss or reduced ARSA function results in the accumulation of sulfatides, particularly in brain white matter, where it leads to myelin damage and progressive loss of motor and cognitive functions [27]. The symptoms vary depending on residual ARSA activity and are used to classify MLD subtypes based on the age of onset. Late‐infantile MLD is the most severe form with the lowest residual ARSA activity, followed by early‐ and late‐juvenile MLD, while adult MLD is the mildest form. Despite remaining uncertainties, the strong genotype–phenotype correlation, the genotype, residual activity of the ARSA enzyme, and sulfatide levels are used to predict the age of onset in MLD, which is a prerequisite for effective treatment decisions [28]. As with X‐ALD, treatment options for MLD remain limited and include gene replacement through HSCT or gene therapy.

For X‐ALD, onset and progression of CALD are currently determined by regular brain MRI. Staging of brain lesion severity and, thus, determining eligibility for HSCT/gene therapy treatment involves the MRI‐based Loes score, a semi‐quantitative 34‐point system developed for X‐ALD comprising visual interpretation and scoring based on both location and extent of cerebral demyelination and atrophy in the brain [29]. However, the MRI screening has its limitations, such as anesthesia in young children and repeated administration of gadolinium (Gd) contrast agents. Further, MRI‐based clinical decision‐making remains challenging for patients with atypical lesion patterns or advanced CALD.

Despite increasing numbers of X‐ALD patients being identified through newborn screening and improved diagnostic procedures, no blood‐based biomarker has been implemented to complement current diagnostics for the onset and progression of CALD in the clinical management of X‐ALD patients. Further, no molecular marker is available in the clinics to predict the risk of onset or increased burden of CALD for individual X‐ALD patients. The disease course of X‐ALD remains highly unpredictable, and a detailed understanding of the underlying mechanisms and the vulnerability of different neural cell types is still lacking. Therefore, identifying and translating biomarkers reflecting neurodegeneration and onset of neuroinflammation in X‐ALD patients is of utmost importance to enhance our understanding and prediction of disease progression and act as a surrogate for clinical outcome measures.

A blood biomarker could be used to detect the initiation of CALD in X‐ALD patients in its earliest stages, possibly at the same time or even before currently used MRI scans involving Gd‐based contrast agents. As a result, early diagnosis of CALD could be improved with increasing numbers of patients having access to life‐saving HSCT or gene therapy, while reducing frequent Gd exposure. A biomarker for X‐ALD could further provide a readout of therapeutic interventions or future clinical trials in X‐ALD, thus directly speeding up the development of alternative therapeutic strategies. By complementing existing evaluation strategies, such a biomarker could improve current MRI‐based assessments, leading to more accurate predictions of the neurological status and, consequently, the quality of life of CALD patients after HSCT or gene therapy. This would especially support decision‐making in advanced CALD cases, for which the success of treatment with survival without major functional disabilities is questionable. In CALD patients with atypical lesion patterns, a biomarker could more precisely reflect neuronal damage and thus provide a more reliable indication of poor outcomes after HSCT. Finally, in AMN, clinical trials are currently hampered by the slow progression rate of the axonal damage and day‐to‐day variation of disease symptoms that obscure the efficacy readouts within the typical time frames of 6 months to 2 years. Here, a biomarker would enable detailed monitoring of the onset and progression of the myelopathy, thus constituting a screening tool for accurate detection and quantification of axonal damage. The advantage of a blood biomarker for X‐ALD patients would be that the blood could be easily taken at local medical institutions at tight intervals and then sent for further analysis to specialized centres. This would limit the travel of X‐ALD patients, which is especially of concern for AMN patients suffering from postural instability and gait dysfunction.

## 4 Blood Biomarkers Reflecting Neurodegeneration and Glial Damage in More Common Disorders

Several blood‐based biomarkers indicative of dysfunction or damage of various neural and barrier cell types have recently emerged. One of the most promising blood biomarkers reflecting neurodegeneration is neurofilament light chain (NfL), a scaffolding protein located primarily within myelinated axons. Upon neuronal damage, NfL is released into the CSF and further into the bloodstream where it can reliably be measured by fourth‐generation immune assays such as Simoa. In contrast to neurofilament heavy chain (NfH) capturing chronic axonal damage, NfL release mirrors acute axonal damage determined by inflammation [30, 31]. Whereas the precise distribution of NfL throughout the CNS and the dynamics of NfL turnover are still matters of research, a study by Kalm and colleagues revealed that NfL concentrations in the blood are not constrained by BBB permeability [32]. NfL is indicative of axonal damage irrespective of cause. Hence, increased blood NfL levels have been shown for various common neurodegenerative disorders, including AD and MS, as reviewed in [33] but also for rare brain disorders such as MLD [34] or X‐ALD [35, 36, 37, 38], as discussed below. With the amount of NfL released into the periphery being associated with the intensity of recent or ongoing neurodegeneration [39], higher blood levels indicate accelerated disease progression and a higher rate of brain atrophy [33, 40]. Recent research suggests that NfL could also be employed for prognostic purposes. A study on autosomal dominant AD demonstrated that the rate of change in blood NfL levels starts to rise as early as 15 years before the onset of symptoms [40]. In MS, studies indicate that blood NfL levels might forecast poorer long‐term clinical and MRI outcomes [39, 41, 42, 43, 44]. Further, increased serum NfL levels correlate with the risk of MS patients developing Gd‐enhancement, indicative of BBB disruption and new T2‐weighted brain lesions within the following year. Notably, in MS serum NfL peaked within a three‐month window surrounding the appearance of Gd + lesions [45, 46]. NfL scores adjusted for age and body mass index allowed the identification of MS patients at greater risk for a detrimental disease course or suboptimal therapy response [45]. Several additional studies have consistently documented the decrease of blood NfL following the initiation of disease‐modifying treatment at the group level, and short‐term changes in NfL levels have been correlated with long‐term MRI studies and clinical outcomes [47, 48, 49, 50, 51, 52].

Next to NfL, the astrocytic intermediate filament glial fibrillary acidic protein (GFAP) shows great potential for diagnosing and monitoring brain disorders, as well as serving as a surrogate endpoint in treatment trials [53]. In the brain, GFAP is expressed by mature astrocytes in both grey and white matter. Of note, GFAP is also present in peripheral cells such as Schwann cells, enteric glial cells, or hepatic stellate cells, although at a much lower extent. As for NfL, GFAP levels in the blood also lack the specificity to diagnose specific brain disorders. Thus, increased blood GFAP levels are common for various neurologic disorders and different states of brain injury, including traumatic brain injury, MS, neuromyelitis optica spectrum disorder (NMOSD), or ad, [54, 55, 56, 57] but also at group level for MLD and X‐ALD [34, 36, 37], as discussed below. In MS, multiple studies identified an association between GFAP and disease status, with blood GFAP levels correlating with expanded disability status scale (EDSS) score [53]. Thus far, the potential of GFAP to forecast future relapses in patients with MS and to track the progression of disability over time remains largely unexplored. In NMOSD, increased or high levels of NfL and serum GFAP may mirror disease activity and precede a clinical attack [58].

Plasma and serum lipid analysis have been suggested as valuable biomarkers for many different disorders. A recent study, however, demonstrated in a cohort of autistic patients the strong influencing factors of the microbiome, diet, medication, sex, age, body mass index (BMI) and how difficult it is to narrow down prime genetic factors underlying blood lipid alterations [59]. Although the diagnosis of inherited lipid metabolism disorders such as in X‐ALD and MLD is relatively straightforward, the precise identification of hypomorphic alleles may still be affected by the wide range of confounding factors influencing plasma lipids.

Next to NfL, GFAP, and potentially lipid biomarkers, several pathology‐specific biomarkers such as amyloid beta (Aß) peptides and hyperphosphorylated Tau protein (p‐TAU) can be quantified in blood‐based samples for neurodegenerative diseases such as AD [60, 61].

## 5 Translation of Blood Biomarkers to the Clinics

The translation from a promising candidate biomarker into the clinics is usually a long and complicated path. Following the identification, a blood biomarker's “scientific validity” is demonstrated by confirmatory proof‐of‐principle steps that verify the connection of the biomarker and a biochemical pathway related to a pathological condition. After group‐level comparisons within the early discovery phase, the clinical significance of these results needs to be assessed before proceeding to proof‐of‐concept studies. The clinical significance refers to the practical importance of the scientific finding. It is determined using measures such as confidence intervals and effect sizes [62], thus serving as a tool to quantitatively determine whether the magnitude of the observed difference holds clinical relevance for patients. Subsequently, a tailored assay for measurement of the biomarker is developed to change the methodology used during the discovery phase for a specific, practical, and up‐scalable testing format. By using already established assay platforms such as immunoassays and commercially available good manufacturing practice (GMP) grade raw material, the approval, and thus implementation into clinical settings, can be accelerated. This prototype assay then serves for proof‐of‐concept studies, where the assay's analytical and clinical performance characteristics are assessed.

The analytical performance of a biomarker test is defined by the ability of a device to correctly detect or measure the analyte. At this step, two performance characteristics are essential to support the translation of biomarker discoveries into clinical use: practicability and reliability. To improve practicability, the specimen used for biomarker testing needs to be readily available, thus allowing for routine and as minimally invasive as possible collection. As such, blood would be preferred over CSF for specimen sampling. To improve reliability, it is important to determine the sensitivity of the biomarker to pre‐analytical factors, including sampling time, handling, processing, and storage. For biomarker measurement, the proper method needs to be critically chosen while taking into account quality control parameters such as precision of measurement and repeatability (such as within‐run, between‐run, between day), limit of detection, limit of quantification, interference, linearity, and the range of measurement. For a biomarker to be robust and scalable, minor measurement errors and pre‐analytical errors should not drastically impact the classification of patients. Blood biomarkers such as NfL and GFAP are reliably quantified in serum and plasma samples using the fourth‐generation immunoassay Simoa. Simoa relies on magnetic beads conjugated with antibodies, which are pulled down into microwells with target analyte and enzyme‐labelled detector antibody to allow for counting individual molecules. Quality control checkpoints recently defined for blood NfL measurement by Arslan et al. include measuring calibrators and samples at least in duplicates, achieving intra‐assay coefficients of variability (CV) below 10%, including three pre‐characterized control samples with low, medium, and high NfL concentration into each run, inter‐assay CVs below 10%, avoidance of different assay versions and announcement of different LOTs and inter‐LOT CV in the method section, and performing the measurements blinded [63]. Proper handling and storage of samples is essential. Existing studies provide guidance on the thawing process and the reuse of serum samples for NfL measurement [64, 65].

The clinical performance of a candidate blood biomarker is defined by its capacity to accurately identify patients with particular clinical conditions or physiological states. As such, it describes the ability to associate with an intended condition within a target population, thereby positively affecting the treatment of patients. Criteria used to assess the clinical performance of a biomarker assay include, next to ROC curves and AUC, more clinically relevant measures such as positive predictive and negative predictive values, which both depend on the prevalence of the disease in the population. The probability of a patient being at risk at a specific biomarker concentration could be estimated using predictiveness curves as visualization aids to understand and evaluate the performance of a predictive model, as introduced by Pepe et al. [66].

A major limitation in assessing the clinical performance of a candidate biomarker is evaluating the biomarker in pooled populations that are not representative of the intended use population. This is especially true for rare disorders where sample size limitations apply. Here, further assessment of the value of combining the candidate biomarker with other factors is needed to reduce the risk of misclassifying patients. Another confounding factor is the development of cut‐off levels for abnormal biomarker concentrations based on highly selected groups of participants that do not consider parameters that might influence the normal reference limits, such as age, BMI, renal or hepatic functions, or disease‐associated comorbidities. A way to deal with this problem is using statistically robust reference databases based on percentiles and *Z* score values to derive reference values corrected for potential confounding factors such as age and BMI, as recently published for serum NfL in large cohorts of pediatric and adult populations [45, 67].

From the long list of candidate biomarkers, only a few can be transformed into in vitro diagnostic device (IVD) biomarker assays. Based on the diagnostic test results of IVD assays, the health status of a person is evaluated, and patients may receive medical care, making the reliability of these tests of critical importance. Accordingly, IVD tests for clinical use are subject to stringent regulatory processes with detailed evaluation of the scientific evidence supporting both the safety and effectiveness of the test before being approved as IVD tests by organizations such as the Food and Drug Administration (FDA) in the United States or certified under the In Vitro Diagnostic Regulation (IVDR) by notified bodies in the European Union. Unlike IVD tests, research‐only products are not subject to these strict regulations and, therefore, are not legally recognized as diagnostic tools, despite the fact that they potentially offer clinically relevant information.

The clinical utility of an IVD biomarker assay is the ability to positively influence the clinical outcome when introduced into the clinical caretaking of patients through screening, monitoring, diagnosis, or patient management. However, when applying a biomarker test, the harms of testing need to be considered, including the risk of false positive or false negative results or the lack of an effective treatment for a specific disorder. As such, the critical criteria for an IVD‐applicable biomarker are that it (a) addresses a significant unmet medical need or replaces an unsatisfying existing solution, (b) classifies patients either based on current or future disease status or treatment outcome, (c) facilitates therapeutic decisions with a low risk of under‐ or over diagnostics, and (d) can be quantitatively measured in minimally invasive sample specimens using practical assays that do not require complex result interpretation. Further, to be introduced into clinical routine, there must be strong evidence for patients´ benefit and the ability to integrate the biomarker test into the routine workflow and run it at affordable costs.

## 6 Current Application of Blood Biomarkers for Brain Pathologies

Fluid biomarkers such as NfL, GFAP, or AD‐specific Aβ peptides and p‐Tau indicate neurodegeneration and/or glial damage in the blood samples. However, for AD, the CSF assay determining the ratio p‐Tau181 to Aβ42 (P‐tau181/Aβ42) remains the only FDA‐approved test for AD‐indicative neurodegeneration associated with cognitive impairment. For MS, a blood test measuring the levels of NfL developed by Siemens Healthineers has recently received the CE (Conformité Européenne) marking of the European Union, certifying that it complies with European Union standards for medical devices. Applicable to MS patients with relapsing disease aged 19 to 55, the test is intended to be used alongside clinical and imaging data to predict which patients are most likely to display signs of new or worsening disease activity over a two‐year follow‐up period. According to recently published guidelines for the use of NfL in the management of MS patients, regular monitoring of blood NfL, in conjunction with other measures of MS severity or prognosis such as MRI, is recommended to determine the change from baseline and to predict the risk for disease worsening, clinical change, and development of Gd + brain lesions [68]. During periods of perceived clinical quiescence, the panel recommends analyzing serum NfL at 3‐ to 6‐month intervals. If a patient experiences clinical worsening and/or MRI changes, elevated serum NfL levels might indicate the need for further examination and consideration of alternative disease‐modifying therapies [68]. Thus, serum NfL may offer a minimally invasive and cost‐effective way to assess the disease status in MS patients with relapsing disease. However, the full implementation of NfL in the decision‐making of clinicians requires robust reference ranges based on normative data adjusted for relevant confounders such as age and BMI [68]. Further, cut‐off points applied in clinical use will require frequent updates as they are often not defined in clinically representative populations of patients [69], with blood samples analyzed in batches resulting in less variability when compared with prospective testing of individuals in clinical caretaking.

## 7 Implementation of Blood Biomarkers in Clinical Caretaking of Patients With Rare Disorders—Lessons to Be Learned for X‐ALD

**FIGURE 2:**
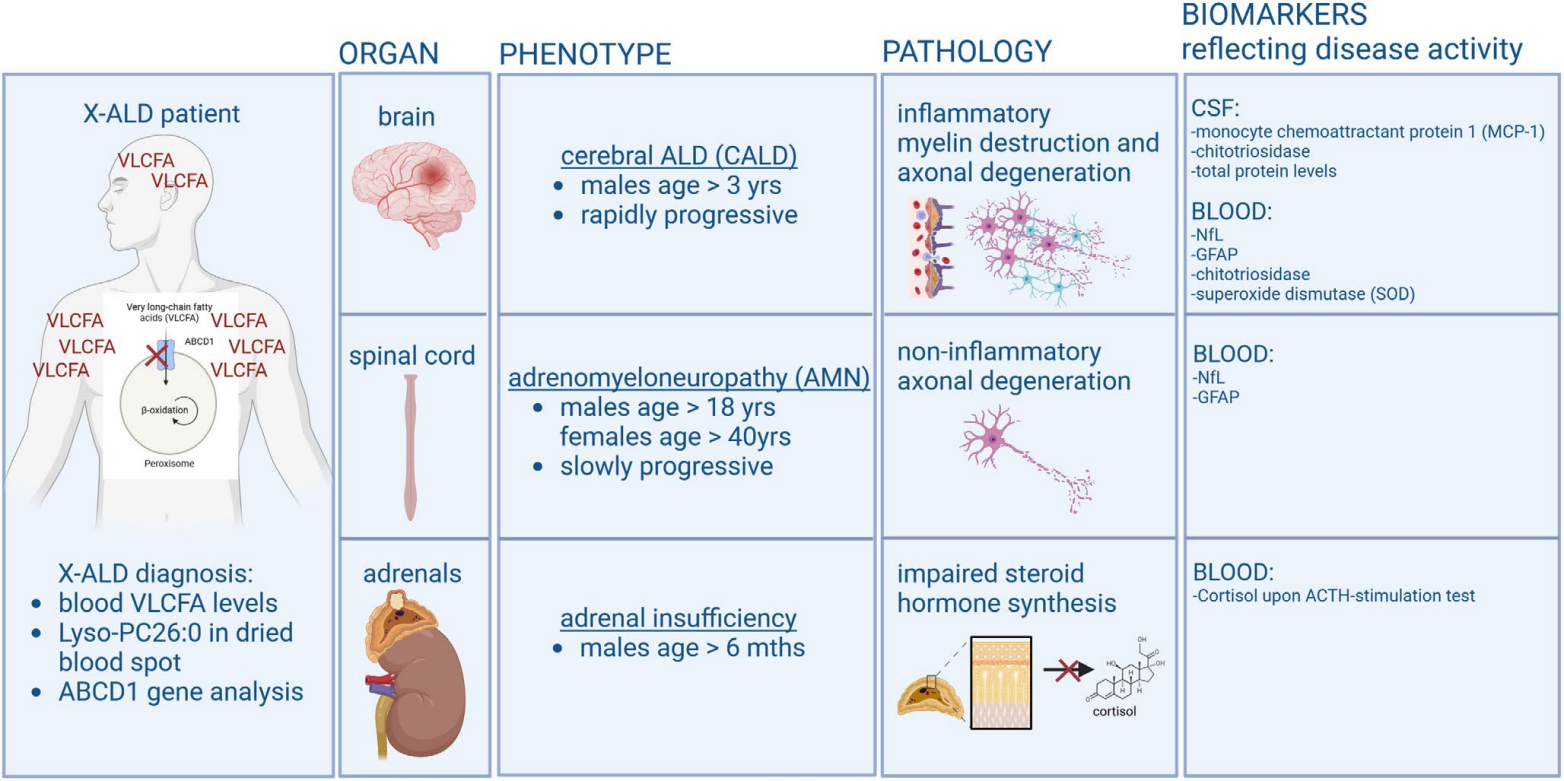
Biomarkers reflecting different X‐ALD related pathologies at the group level. The diagnosis of X‐ALD is performed by the detection of saturated very long‐chain fatty acids (VLCFA) in the lyso‐PC fraction (right panel). Biomarkers indicative of cerebral inflammation, spinal cord axonal degeneration, and adrenal insufficiency are depicted.

How well do blood biomarkers so far represent X‐ALD pathology? Over the last years, several efforts have been made to establish a blood biomarker for X‐ALD that would correlate with the onset, progression, and severity of the disease. A thorough compilation of the different candidate markers identified so far linked to neuropathologic hallmarks of the disease, including disturbed VLCFA metabolism, inflammation, and oxidative stress, as well as neuro‐axonal and astroglial damage, is presented within a recent excellent review by Honey et al. [70]. A schematic representation summarizing the neuropathological features of AMN and CALD and their association with key candidate blood biomarkers is depicted in Figure 2. Here, we will mainly focus on recent advancements in X‐ALD research on NfL, which shows high promise as a blood biomarker to indicate neuronal damage and to reflect the activity and progression of the neuroinflammatory CALD course in X‐ALD patients.

For GFAP, increased blood levels were observed not only in symptomatic X‐ALD but also in MLD patients at group level [34, 36, 37]. In both X‐ALD and MLD, GFAP levels correlate with symptom severity. As discussed below for NfL, GFAP is increased to a much lesser extent in the adult, slowly progressive form of MLD or the adult CALD form than in the rapidly progressive late‐infantile MLD or the childhood form of CALD. In MLD, this also appears to be true when comparing slowly progressive patients with late‐juvenile or adult MLD to those with rapid progression. In late‐juvenile MLD, a significant difference is observed, while in adults, GFAP values are comparable between the two groups. Similarly, in X‐ALD, lesion intensity with regard to the Loes score is comparable between the two cohorts [36]. Taken together, blood GFAP levels indicative of astrocyte activation or damage might have additional value for addressing more specific clinical questions, but in general, NfL currently appears to be the preferable marker. For X‐ALD, the combination of GFAP and NfL did not further improve the ability of NfL to discriminate inflammatory CALD from non‐inflammatory X‐ALD in paediatric patients [36].

NfL only indirectly reflects the inflammatory aspect of neuroinflammation, as recently proposed for MS [30]. So far, no peripheral inflammatory factor such as a chemokine or cytokine has yet been identified to indicate CALD. Highly interesting, blood cytokine levels of IL‐15, CCL7, and IL‐12p40 were already significantly increased in the pre‐symptomatic X‐ALD children when compared to the age‐matched control group on the group level. These inflammatory markers did not further increase in children with the onset of CALD [36]. Nevertheless, such markers might be of value for further characterisation of ABCD1 variants of uncertain significance.

Based on group‐level comparisons, NfL was found by three studies to be modestly but significantly increased with slowly progressive non‐inflammatory myelopathy in AMN patients [35, 36, 37]. Upon conversion to the neuroinflammatory CALD phenotype, NfL levels rose sharply, reflecting inflammatory activity and progression of neuronal damage and myelin loss [35, 36]. Importantly, when longitudinally comparing NfL levels in disease progression, it needs to be considered that “burned out lesions”, as observed in late‐stage CALD patients with a very high MRI severity score and a long period in the vegetative state, could result in relatively low NfL levels that are within the range of healthy controls (personal unpublished observations). Recently, Golse et al. demonstrated a statistically significant dependence of blood NfL on the total lesion volume in CALD patients, indicating that NfL is a dynamic marker of axonal damage that correlates in X‐ALD with brain lesion volume [21]. In support of these findings of NfL in the context of CALD, a study carried out by Wang and colleagues focusing on plasma samples from pediatric CALD patients and using another methodological approach (Olink versus Simoa) could confirm the increase of NfL in the presence of neuroinflammation [38].

**FIGURE 3:**
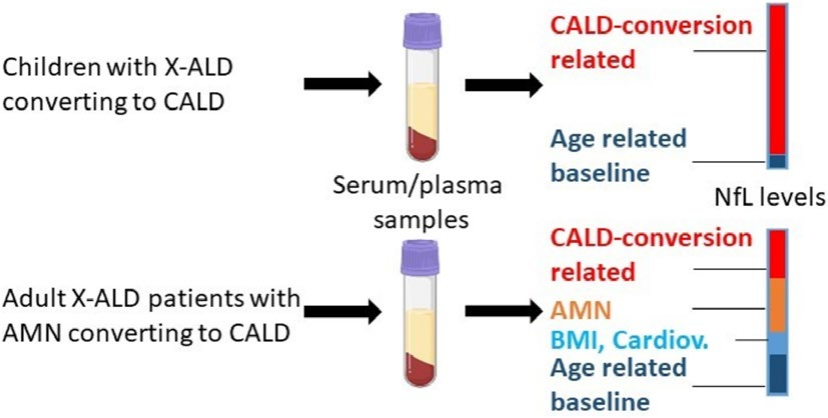
The NfL levels in children and adult patients with X‐ALD are affected by various factors that complicate the detection of early conversion to the cerebral form of X‐ALD in adult patients. NfL levels are age‐dependent with a U‐shaped characteristic, having the lowest levels between 10 and 18 years. Thus, in children, the baseline background is lower than in adult patients (dark blue). NfL levels are higher in CALD children than in adult with CALD despite similarly graded MRI Loes‐scored lesion severity. In adult X‐ALD patients suffering from non‐inflammatory AMN, NfL levels are increased compared to age‐matched healthy controls (orange). Whereas in both children and adults, NfL levels are affected by age as a confounding factor, the amount of blood NfL is further impacted by additional factors such as BMI or cardiovascular disease in adults, thus resulting in a higher NfL baseline variability in adults when compared to children.

Of note, when plasma NfL levels from children and adults with cerebral ALD with a similar distribution of MRI Loes‐scored brain lesions status were compared, significantly lower blood NfL levels were found in adults than in childhood CALD patients [36]. The lower CALD‐related increase in NfL levels occurs upon a higher baseline NfL background in adult X‐ALD men due to the myelopathy present in most adult male X‐ALD patients. Since NfL is measured as the concentration of an analyte within a defined volume of plasma or serum, age‐ and body weight‐related differences in blood volume could further contribute to the observed differences in NfL levels between pediatric and adult CALD patients. Accordingly, these factors contribute to the challenges of using NfL as a predictor for the onset of the cerebral form in adult AMN patients (Figure 3). In support of this notion, studies reported that people with increased body‐mass‐index and therefore higher blood volume present with decreased blood NfL [71, 72]. As the inverse correlation of blood NfL with blood volume was not observed for CSF NfL levels [71], it might be interesting to directly compare CSF from childhood and adult CALD patients regarding their amount of NfL.

In MLD, where, in contrast to X‐ALD, a genotype–phenotype correlation is present, NfL is strongly increased in symptomatic MLD patients when compared to controls, with the increase of NfL being associated with reduced activity of the ARSA enzyme and, consequently, with a more severe phenotype and more rapid progression [34]. The lowest increase in NfL was observed in adult MLD [34]. However, the value of blood NfL levels for phenotype prediction in pre‐symptomatic patients remains unresolved [73]. This is largely because NfL only indicates ongoing axonal damage, meaning that baseline levels in asymptomatic patients cannot be predictive until the acute phase of brain damage begins. Therefore, a key focus will be determining how early the initial increase in NfL levels occurs in relation to the first MRI alterations and clinical symptoms.

The ability to classify the presence of CALD in adult CALD patients is further constrained by the occurrence of a smoldering disease course in a subset of patients [35]. In these patients diagnosed with ongoing CALD and graded with MRI brain lesion severity Loes scores ≥ 3, only marginally elevated NfL levels were observed when compared to the highly elevated levels in other adult CALD patients with similar MRI scores. Of note, the patients presented with only hazy Gd‐enhancement in brain MRI possibly indicating a low‐grade neuroinflammatory process occurring behind an only partially disrupted BBB [35]. In people with relapsing MS, plasma NfL levels peak during the inflammatory relapse phases, thus reflecting increased axonal damage during the inflammatory activity of the disease course. In contrast, during later stages or in progressive MS, the NfL levels in the blood show a steady increase similar to normal aging, albeit at higher levels than in controls [74]. Therefore, in MS, blood NfL levels seem not to accurately capture the progression of disability in the absence of acute inflammation [30, 74]. Somewhat similar to progressive MS, the smoldering disease activity present in a subset of adult CALD patients and characterized by only modestly increased blood NfL might include such low‐grade inflammation composed of infiltrating and CNS‐resident activity of myeloid cells together with neuronal deregulation and damage that is reflected by a slow chronic deterioration over time [30]. Thus, detecting the conversion of X‐ALD patients with myeloneuropathy (AMN) to the neuroinflammatory CALD poses a significant challenge due to the lack of specificity in clinical symptoms and the low sensitivity of the MRI Loes score in the early stages of the disease course [75]. With merely one‐third of adult CALD patients currently being identified early enough for eligibility to HSCT, there is an urgent need to develop new biomarker strategies that reliably indicate early‐stage neuroinflammation in adult X‐ALD patients. Here, instead of relying on a single biomarker, novel approaches might leverage comprehensive molecular omics and multi‐omics interrogation techniques for multi‐analyte detection.

Blood NfL is a dynamic biomarker with a documented decrease upon the initiation of disease‐modifying treatment in neurodegenerative disorders such as relapsing MS [47, 48, 49, 50, 51, 52]. With reduced blood NfL associated with improved clinical and MRI outcomes, NfL is a reliable disease‐monitoring biomarker in relapsing MS. In X‐ALD, determining NfL in CALD patients at baseline and over time upon HSCT or gene therapy may offer a more sensitive measure of treatment effectiveness than clinical or MRI measures alone. Additionally, the assessment of blood NfL before and after treatment could in future, when pre‐treatment NfL levels have been associated with treatment outcome in a larger cohort, help to evaluate if baseline NfL levels in CALD patients would be predictive of treatment outcome. Predicting the expected neurological status and, therefore, quality of life of CALD patients after receiving HSCT or gene therapy could be especially advantageous for patients with atypical brain lesion patterns, for whom the outcome of these interventions is often uncertain. After HSCT in CALD patients, the myelin destruction, as assessed by brain MRI, is usually continuous for up to 1 year, whereas BBB repair and donor cell recovery reflected by blood neutrophil count is usually observed within the first 100 days post‐treatment [76]. This suggests that successful engraftment of donor cells within the brain of CALD patients requires weeks to months after HSCT/gene therapy treatment, leading to expected delays in the reduction of NfL. In a small‐scale pilot study, Weinhofer et al. could show that blood NfL gradually normalized and approached control levels in adult CALD patients who had received HSCT [35]. In accordance with MRI data revealing prolonged myelin destruction up to 1 year after treatment, the data suggested that decreases in NfL would not be apparent until 1‐year post‐HSCT [35]. These findings were confirmed by Wang and colleagues, who investigated 11 pediatric CALD patients before and up to 1 year after HSCT [38]. Future prospective studies with larger cohorts of CALD patients and more frequent and extended longitudinal sampling before and after treatment are needed to determine the duration of ongoing neuronal injury and myelin loss before the treatment finally halts the damage. This knowledge would also be valuable for the development of alternative therapeutic approaches targeting neuronal/axonal integrity combined with the halt of neuroinflammation.

Next to predicting and monitoring treatment outcomes, a major goal is to evaluate whether blood NfL could be used as a tool to support the classification of early‐stage CALD in X‐ALD children. Especially given the fact that the increase in NfL seems to occur rapidly and in MS correlates with the risk of developing Gd‐enhancement, this biomarker holds promise for early detection of CALD in X‐ALD patients [45, 46]. However, given the rarity of X‐ALD with a combined male and female incidence 1:14.700 births, and 1/3 of boys below the age of 12 developing CALD [7], the translation of NfL from the proof‐of‐principle steps outlined above toward proof‐of‐concept studies with a detailed assessment of both analytical and clinical performance cannot be addressed by a single center at a national level but needs a combined international effort of collaborating X‐ALD centers. This further development of NfL for clinical use includes documented performance of NfL to accurately identify early‐stage CALD at the individual level, requiring validation in prospective intended‐use cohorts of X‐ALD children preferentially recruited from newborn screening programs and monitoring of siblings. In a first effort toward this end, Weinhofer et al. previously determined a pediatric CALD‐indicative plasma NfL cut‐off value [36]. The CALD‐indicative plasma NfL cut‐off value, defined within a discovery cohort of 41 CALD and 20 asymptomatic X‐ALD children, enabled correct classification of the cerebral status in 24 out of 25 X‐ALD children of a separate validation cohort [36]. Despite this high accuracy, two limitations need to be addressed. Firstly, the validation cohort of Weinhofer et al. consisted of a selected population of boys with X‐ALD: asymptomatic intended‐use patients (*n* = 5), early‐stage CALD (Loes score ≤ 2.5; *n* = 7) but also patients with more advanced CALD (Loes score > 2.5; *n* = 13) presenting highly accelerated NfL values. Secondly, recent work by Abdelhak et al. representing data from 2667 healthy children revealed that due to physiological changes, blood NfL levels are clearly age‐dependent, with serum NfL concentrations decreasing until the age of 10 years followed by a stabilization up to the age of 22 years [67]. Accordingly, a fixed childhood CALD‐indicative NfL cut‐off value may interfere with the sensitivity and specificity of the biomarker test, especially in younger children.

Thus, age‐adjusted CALD‐indicative NfL scores could be defined, and future aims should be addressed to develop a web tool as previously carried out for MS [67] that is accessible for clinicians to determine and evaluate the NfL scores of X‐ALD children derived from standardized NfL measurements. Such a tool would be able to aid the assessment of the risk for CALD onset in individual children, thus potentially increasing the number of boys affected by CALD gaining access to life‐saving HSCT or gene therapy, while possibly concurrently reducing the frequency of strenuous MRI scans.

## 8 Concluding Remarks and Future Outlook

It is exciting that due to novel highly sensitive techniques, blood‐based biomarkers can be reliably used for the quantification of brain pathology, in particular axonal damage, by quantification of NfL. With NfL being indicative of axonal damage irrespective of cause, NfL quantification serves as a diagnostic marker, but also as a prognostic and monitoring marker in many different diseases. The correlation between NfL levels and disease activity makes it a valuable prognostic marker, as recently demonstrated for MS, enabling clinicians to better predict disease trajectory and to tailor treatment strategies accordingly. Thus, regular monitoring of NfL levels could provide insights into disease activity, facilitating adjustments in therapeutic approaches based on the progression rate not only for MS [77] but for a variety of neurodegenerative disorders including inherited metabolic disorders. Initial studies indicate the importance of NfL in X‐ALD. The step from scientific interest to application is imminent for NfL. Standard guidelines for the collection and storage of samples must be pointed out. If all this is fulfilled, NfL can improve the clinical management of X‐ALD and allow an individualized approach. In this case, regular clinical checks and collection of NfL every 3–6 months should be suggested. The broad use of NfL for common neurodegenerative disorders as well as for rare inherited metabolic diseases such as MLD and X‐ALD will enable an accelerated implementation through shared knowledge. With regard to the evaluation of novel therapeutic strategies, the acceptance of NfL as a surrogate biomarker for drug efficacy in amyotrophic lateral sclerosis (ALS) associated with mutations in the superoxide dismutase type 1 (SOD1) gene by the U.S. Food and Drug Administration leading to an accelerated approval of tofersen (marketed as Qalsody) exemplifies the progress [78].

As described, the current methods, in spite of all the advances, still have their limitations. As we are currently experiencing a time of rapid technological advancement, techniques such as epigenetic profiling of blood‐derived cell‐free DNA to detect brain cell death may emerge as valuable future tools for enhancing personalized diagnosis, patient monitoring, and treatment decision‐making.

## Author Contributions

I.W. and J.B. conceived the review; I.W., J.B., and P.R. wrote the original draft of the manuscript; I.W., J.B., and P.R. revised the original draft; I.W. and J.B. generated figures.

## Conflicts of Interest

The authors declare no conflicts of interest.
